# Crystal structure of [Ni(OH_2_)_6_]Cl_2_·(18-crown-6)_2_·2H_2_O

**DOI:** 10.1107/S2056989024010041

**Published:** 2024-10-24

**Authors:** Jacob P. Brannon, Kevin Liang, S. Chantal E. Stieber

**Affiliations:** a Department of Chemistry & Biochemistry California State Polytechnic University, Pomona 3801 W Temple Ave Pomona CA 91768 USA; Harvard University, USA

**Keywords:** crystal structure, nickel, Ni(II), 18-crown-6, crown ether

## Abstract

The crystal structure of the title compound, hexa­aqua­nickel(II) dichloride–1,4,7,10,13,16-hexa­oxa­cyclo­octa­deca­ne–water (1/2/2), [Ni(H_2_O)_6_]Cl_2_·2C_12_H_24_O_6_·2H_2_O, is reported. The asymmetric unit contains half of the Ni(OH_2_)_6_ moiety with a formula of C_12_H_32_ClNi_0.50_O_10_ at 105 K and triclinic (*P*1) symmetry. The [Ni(OH_2_)_6_]^2+^ cation has close to ideal octa­hedral geometry with O—Ni—O bond angles that are within 3° of idealized values. The supra­molecular structure includes hydrogen bonding between the water ligands, 18-crown-6 mol­ecules, Cl^−^ anions, and co-crystallized water solvent. Two crown ether mol­ecules flank the [Ni(OH_2_)_6_]^2+^ mol­ecule at the axial positions in a sandwich-like structure. The relatively symmetric hydrogen-bonding network is enabled by small Cl^−^ counter-ions and likely influences the more idealized octa­hedral geometry of [Ni(OH_2_)_6_]^2+^.

## Abstract 2

A new crystal structure of [Ni(OH_2_)_6_]Cl_2_·(18-crown-6)_2_·2H_2_O is reported, demonstrating the effect a small chloride counter-ion has on the hydrogen-bonding network.

## 1. Chemical context

Crown ethers are common chelating agents that are widely used in organometallic chemistry to encapsulate counter-ions for more facile crystallization (Kundu *et al.*, 2019 ▸; Tondreau *et al.*, 2013 ▸), but crown ethers also have broader applications in materials, sensing, and medicines (Gokel *et al.*, 2004 ▸; Li *et al.*, 2017 ▸). Among the first reports of using a crown ether as a chelating agent for a metal was in 1967, demonstrating that crown ethers can chelate directly to metals *via* the oxygen atoms, as evidenced by shifts in the IR spectra (Pedersen, 1967 ▸). The oxygen atoms on crown ethers can also act as hydrogen-bond acceptors, with some examples of donors being NH_4_^+^ (Akutagawa *et al.*, 2002 ▸), *R*NH_3_^+^ (Pedersen, 1967 ▸; Shinkai *et al.*, 1985 ▸; Sutherland, 1986 ▸; Stoddart, 1988 ▸; Izatt *et al.*, 1995 ▸), *R*_2_NH_2_^+^ (Kolchinski *et al.*, 1995 ▸; Ashton *et al.*, 1997 ▸), and *M*—OH_2_ (Cusack *et al.*, 1984 ▸).

18-Crown-6 has also been shown to stabilize octa­hedral metal complexes *via* hydrogen-bonding networks, for example, in metal nitrate complexes (Junk *et al.*, 1998 ▸). The [18-crown-6][Ni(NO_3_)(H_2_O)_5_]NO_3_·H_2_O complex is reported to have a pseudo-octa­hedral Ni^II^ center, with one nitrate and five water ligands, although the nickel complex was not explicitly discussed in the paper, and the full structural data are not in the Cambridge Structural Database (Junk *et al.*, 1998 ▸). The hydrogen-bonding network is reported to be between water ligands and two neighboring 18-crown-6 mol­ecules, the nitrate counter-ion, and water, at distances ranging from 2.679 (9) to 3.05 (1) Å. Water ligands on Ni^II^ have also been shown to act as hydrogen-bond donors intra­molecularly (Brazzolotto *et al.*, 2019 ▸).

There are few crystallographically characterized systems containing [Ni(OH_2_)_6_]^2+^ and 18-crown-6, with two examples reported in the same study: [Ni(OH_2_)_6_][ClO_4_]_2_·(18-crown-6)_2_·2H_2_O and [Ni(OH_2_)_6_]_3_[NiBr_2_(H_2_O)_4_][Br]_6_·(18-crown-6)_4_·2H_2_O (Steed *et al.*, 1998 ▸). This current work highlights the effect that a smaller Cl^−^ ancillary counter-ion has on the supra­molecular structure and octa­hedral distortion of [Ni(OH_2_)_6_]^2+^ co-crystallized with 18-crown-6.
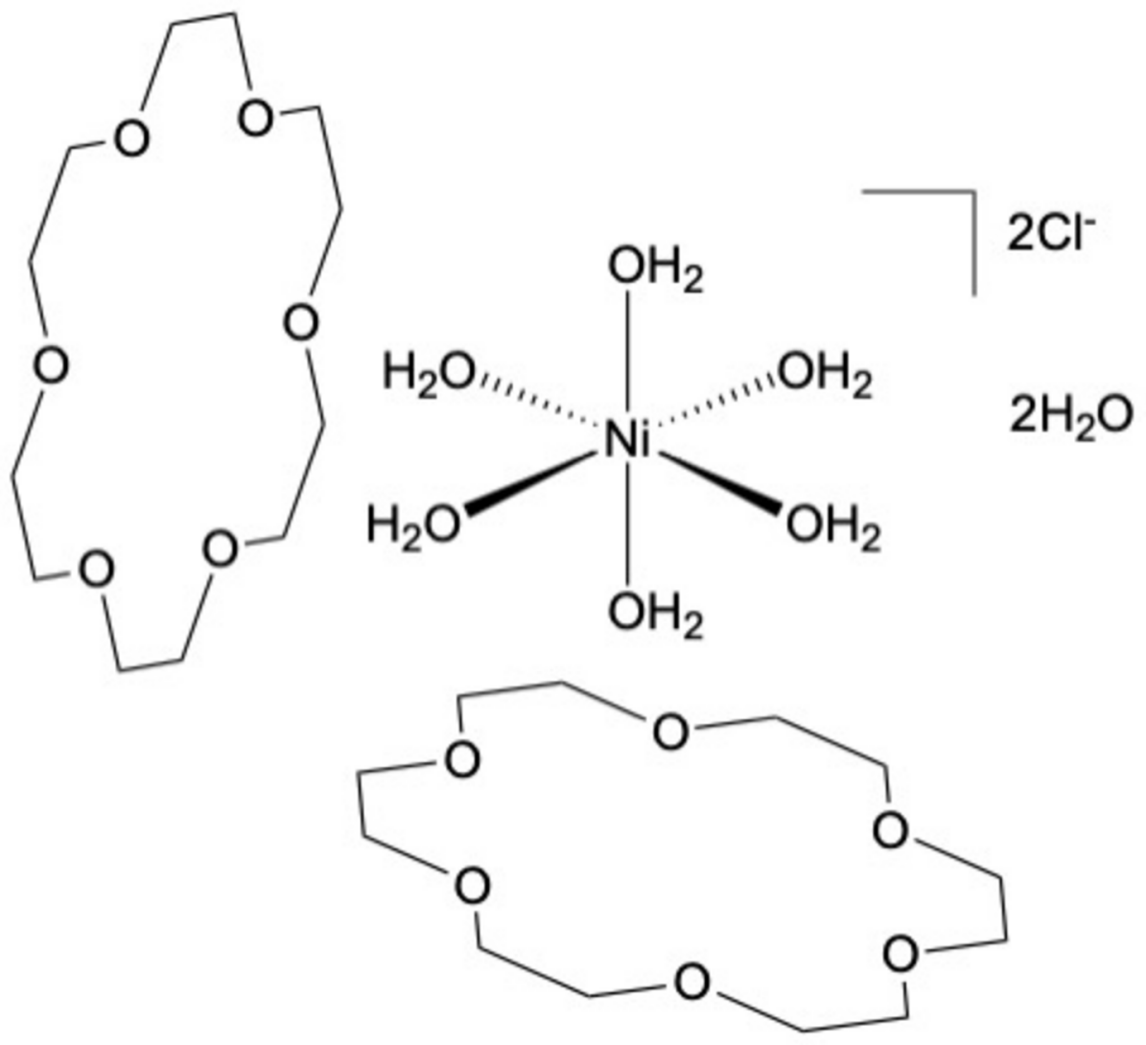


## 2. Structural commentary

**Figure 1:**
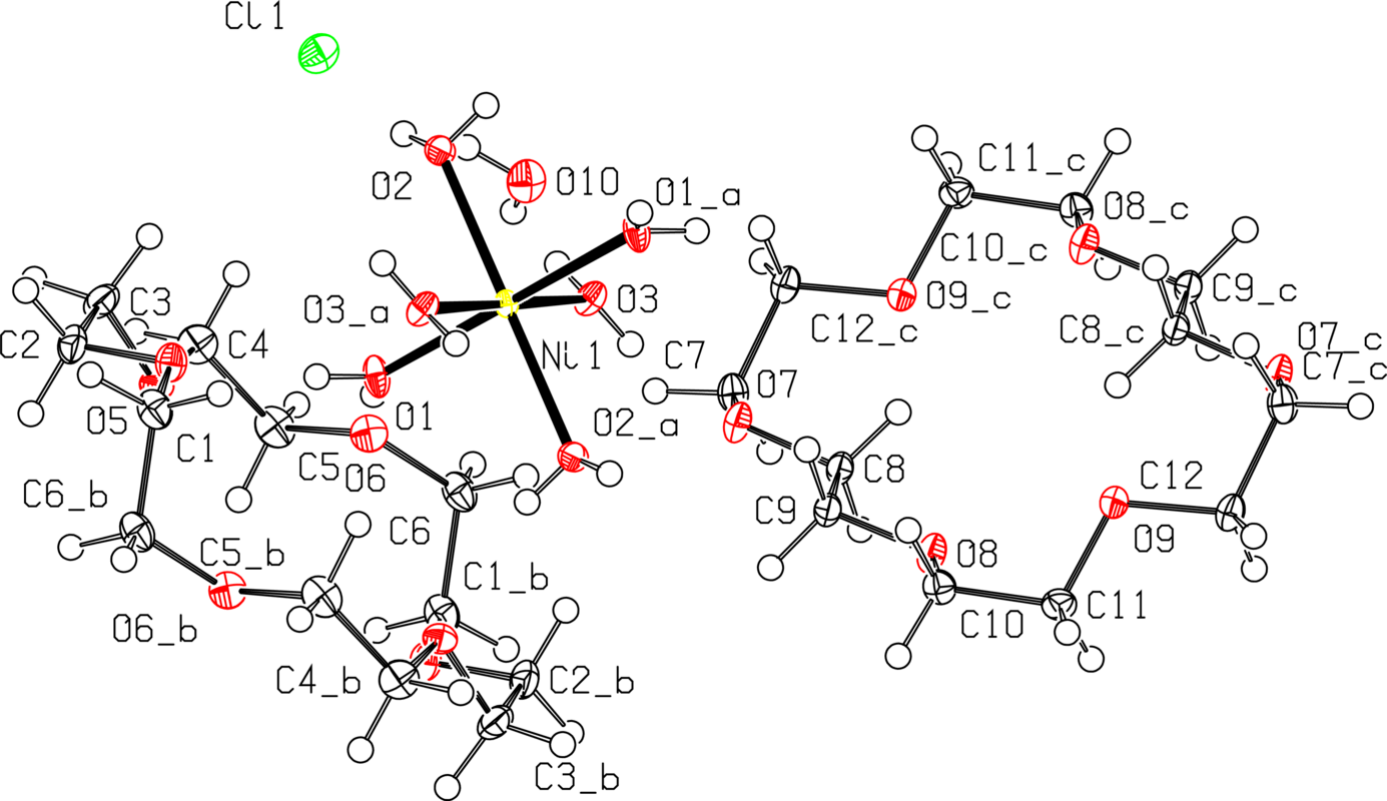
View of [Ni(OH_2_)_6_]Cl_2_·(18-crown-6)_2_·2H_2_O with 50% probability ellipsoids. H atoms are omitted for clarity.

Two asymmetric units make up the structure of [Ni(OH_2_)_6_]Cl_2_·(18-crown-6)_2_·2H_2_O, which has two Cl^−^ counter-ions to balance the Ni^II^ center in [Ni(OH_2_)_6_]^2+^ (Fig. 1 ▸). The [Ni(OH_2_)_6_]^2+^ has close to perfect octa­hedral geometry with O—Ni—O bond angles of 91.62 (3)° for O1—Ni1—O2, 91.05 (3)° for O1—Ni1—O3, and 92.90 (3)° for O2—Ni1—O2. The bond angles for all *trans-*water substituents on nickel are 180° (O—Ni—O), as a result of the triclinic (*P*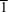) symmetry. This represents a much more symmetric [Ni(OH_2_)_6_]^2+^ cation than the previously reported structure with 18-crown-6, which had *trans* water-ligand angles in the range of 174.43 (7)–178.42 (7)° (Steed *et al.*, 1998 ▸).

The Ni—O bond distances are 2.0310 (8) Å for Ni1—O1, 2.0567 (8) Å for Ni1—O2, and 2.0474 (8) Å for Ni1—O3. These distances are consistent with a slight axial compression for Ni1—O1, but it is not as pronounced as the axial Ni—O distance of 2.0066 (16) Å reported for [Ni(OH_2_)_6_][ClO_4_]_2_·(18-crown-6)_2_·2H_2_O (Steed *et al.*, 1998 ▸).

## 3. Supra­molecular features

**Figure 2:**
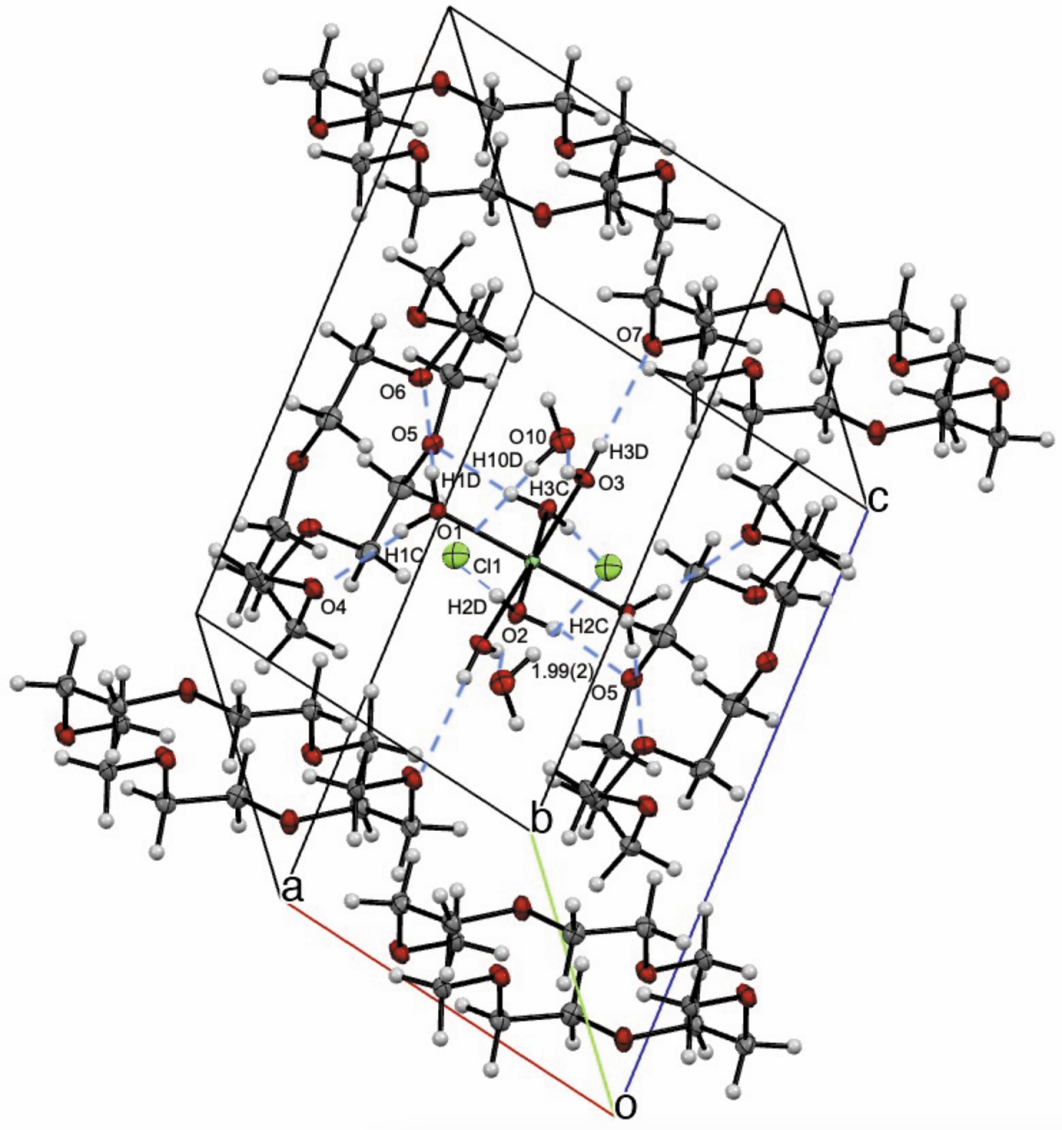
View of the unit cell for [Ni(OH_2_)_6_]Cl_2_·(18-crown-6)_2_·2H_2_O with 50% probability ellipsoids, highlighting inter­molecular distances. Distances including H atoms are listed without standard deviations because the H atoms were positionally fixed. Additional distances are labeled in Fig. 3 ▸ for clarity.

The supra­molecular structure of [Ni(OH_2_)_6_]Cl_2_·(18-crown-6)_2_·2H_2_O is stabilized *via* extensive hydrogen bonding (Figs. 2 ▸ and 3 ▸). The differences in Ni—O bond distances are rationalized by differing hydrogen-bonding inter­actions to each water moiety bound to Ni in the asymmetric unit. The axial water moiety has hydrogen bonding to only 18-crown-6, whereas the equatorial water moieties have hydrogen bonding to 18-crown-6 and chloride or water. The axial water moiety containing O1, H1*C*, and H1*D*, has hydrogen bonding to the neighboring 18-crown-6 mol­ecule with distances of 1.973 (18) Å for O4⋯H1*C* and 1.956 (18) Å for O6⋯H1*D* (Table 1 ▸). By contrast, the equatorial water moiety containing O2, H2*C*, and H2*D*, has hydrogen bonding to the neighboring 18-crown-6 mol­ecule with a distance of 1.991 (15) Å for O5⋯H2*C*, and to one Cl^−^ atom with a distance of 2.335 (19) Å for Cl1⋯H2*D*. The second equatorial water moiety containing O3, H3*C*, and H3*D*, has hydrogen bonding to the neighboring 18-crown-6 mol­ecule with a distance of 2.146 (18) Å for O7⋯H3*D*, and to one water mol­ecule with a distance of 1.84 (2) Å for O10⋯H3*C*. Combined, these differing hydrogen-bonding partners for the H_2_O ligands result in the varying Ni—O bond distances in [Ni(OH_2_)_6_]^2+^. An additional hydrogen bond stabilizes the structure between H_2_O and Cl^−^ with 2.30 (2) Å for H10*D*⋯Cl1.

**Figure 3:**
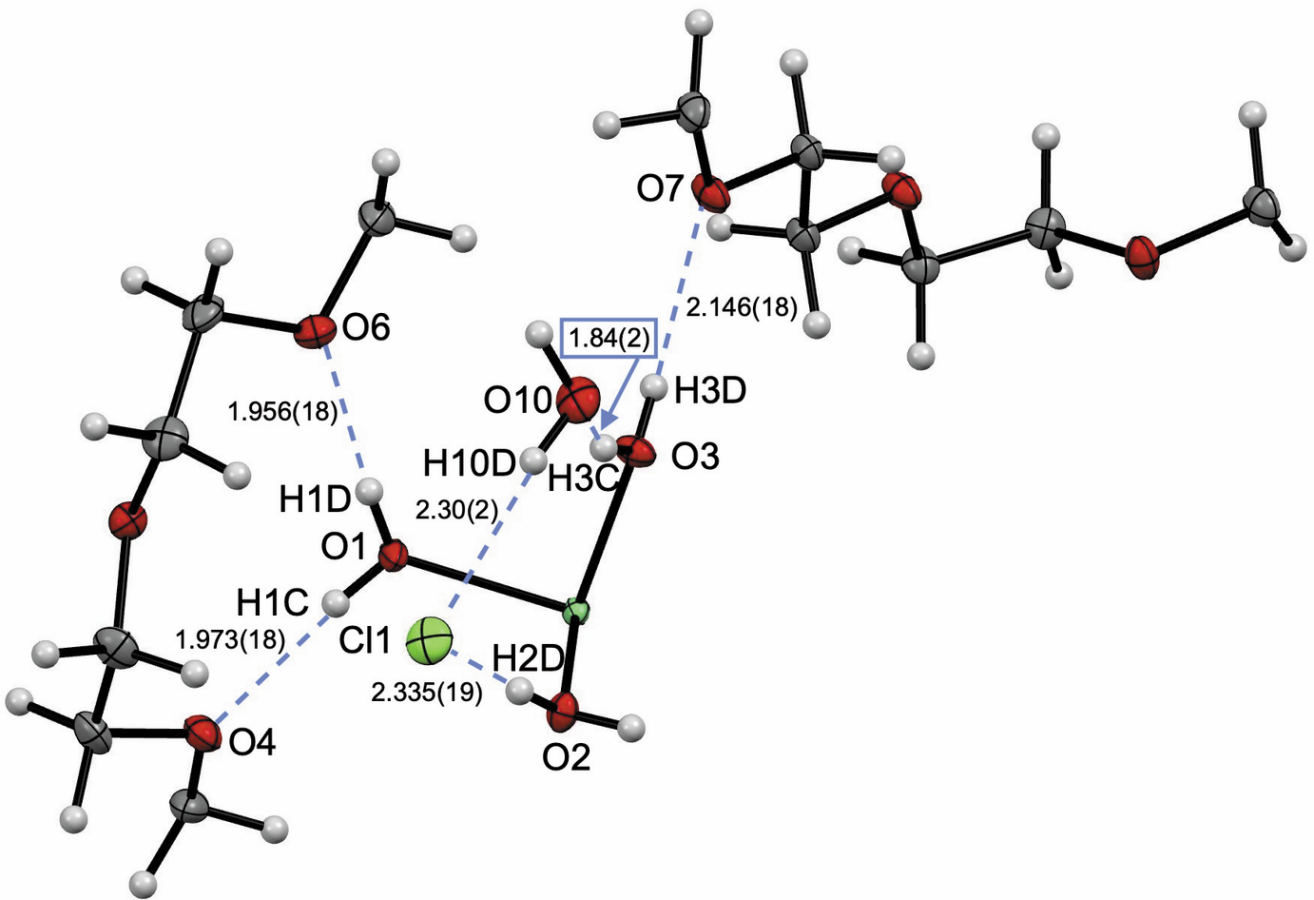
View of the asymmetric unit for [Ni(OH_2_)_6_]Cl_2_·(18-crown-6)_2_·2H_2_O with 50% probability ellipsoids, highlighting inter­molecular distances. Distances including H atoms are listed without standard deviations because the H atoms were positionally fixed.

The significant effect of the counter-ion on the supra­molecular structure and hydrogen bonding is evident from the smaller Cl^−^ counter-ions as compared to the ClO_4_^−^ counter-ions in the previously reported structure (Steed *et al.*, 1998 ▸). The counter-ion size and hydrogen bonding likely influences the [Ni(OH_2_)_6_]^2+^ geometry and level of distortion from octa­hedral symmetry. In both structures, each of the axial OH_2_ moieties forms two hydrogen bonds the neighboring 18-crown-6 mol­ecule (Figs. 3 ▸ and 4 ▸). When Cl^−^ counter-ions are present, one equatorial water forms a hydrogen bond to the top 18-crown-6 mol­ecule, and one equatorial water forms a hydrogen bond to the bottom 18-crown-6 mol­ecule, with both having an additional hydrogen bond each to Cl^−^ counter-ions. The other two *trans* equatorial water ligands have hydrogen bonds to additional neighboring 18-crown-6 mol­ecules and a water mol­ecule each. The ^1^H NMR spectrum in CDCl_3_ suggests that at least some of the supra­molecular structure is maintained in solution, with two proton signals at 3.52 and 3.58 ppm, assigned to the equatorial water ligands due to lack of HSQC or HMBC carbon correlations. This is significantly shifted from the expected shift for free water in CDCl_3_ at 1.56 ppm (Babij *et al.*, 2016 ▸), and is consistent with a previous NMR and crystallographic study of {[(CH_3_)_2_SnCl_2_·H_2_O]_2_·18-crown-6}_*n*_, where water ligand hydrogen bonding to 18-crown-6 was maintained in non-coordinating solvents (Amini *et al.*, 2006 ▸). The overall structure is therefore relatively symmetric with minimal distortion to the octa­hedral symmetry of [Ni(OH_2_)_6_]^2+^, and NMR data suggest that hydrogen bonding to the 18-crown-6 mol­ecule is preserved in deuterated chloro­form solvent.

**Figure 4:**
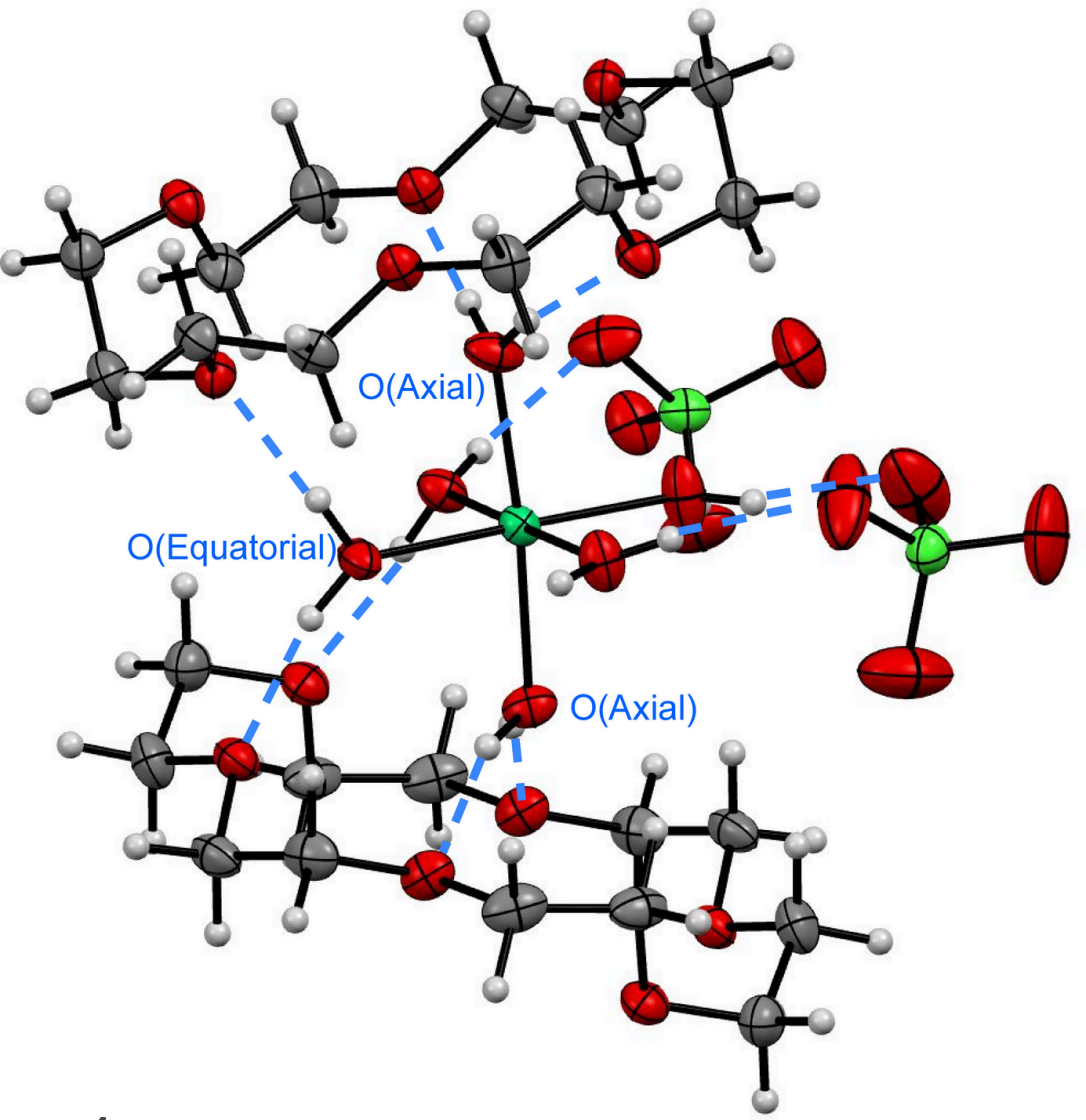
View of [Ni(OH_2_)_6_][ClO_4_]_2_·(18-crown-6)_2_ (Steed *et al.*, 1998 ▸), highlighting hydrogen bonding from axial and equatorial water ligands.

The structure for [Ni(OH_2_)_6_][ClO_4_]_2_·(18-crown-6)_2_·2H_2_O is much less symmetric at a supra­molecular level (Fig. 4 ▸), which is attributed to the ClO_4_^−^ counter-ions (Steed *et al.*, 1998 ▸). One equatorial water ligand forms a hydrogen bond to each of the top and bottom 18-crown-6 mol­ecules, resulting in those mol­ecules being brought closer to each other on one side. The flanking *trans* equatorial water ligands each form a hydrogen bond to a neighboring 18-crown-6 mol­ecule, and a second hydrogen bond to a ClO_4_^−^ counter-ion. This less symmetric network of hydrogen bonding results in stronger distortions in both the Ni—O bond lengths and O—Ni—O bond angles, as compared to the structure with Cl^−^ counter-ions.

## 4. Database survey

The Cambridge Structural Database (Groom *et al.*, 2016 ▸) has almost 400 structures containing a Ni(OH_2_)_6_ moiety; however, only two reported structures were found that contain 18-crown-6 (Web accessed June 3, 2024). The two reported structures are [Ni(OH_2_)_6_][ClO_4_]_2_·(18-crown-6)_2_·2H_2_O and [Ni(OH_2_)_6_]_3_[NiBr_2_(H_2_O)_4_][Br]_6_·(18-crown-6)_4_·2H_2_O (CSD Nos. 113101 and 113105; Steed *et al.*, 1998 ▸). By contrast, there are 64 reported structures in the Cambridge Structural Database that contain a Ni(OH_2_)_6_ moiety with 15-crown-5 (Web accessed June 3, 2024).

## 5. Synthesis and crystallization

**General considerations.** All reagents were purchased from commercial suppliers and used without further purification. ^1^H and ^13^C NMR data were collected on a Varian 400 MHz instrument and referenced to residual CHCl_3_ (7.26 ppm). Full NMR data can be accessed through Zenodo (Brannon & Stieber, 2024 ▸).

**Synthesis of [Ni(OH_2_)_6_]Cl_2_·(18-crown-6)_2_·2H_2_O.** A scintillation vial was charged with 0.025 g (0.19 mmol, 1 eq.) of NiCl_2_ to 0.105 g (0.386 mmol, 2 eq.) of 18-crown-6 ether in 10 mL of tetra­hydro­furan or aceto­nitrile. The vial was heated to 353 K for 1.5 h and placed in a 277 K fridge to cool for 1 week. After 1 week, the cap was removed for slow evaporation over 5 days, resulting in a non-crystalline light-blue solid. The solid was taken into deionized water and light blue crystals suitable for X-ray diffraction were obtained after 2 months in a 277 K fridge and identified as [Ni(OH_2_)_6_(18-crown-6)_2_]Cl_2_·2H_2_O. ^1^H NMR (CDCl_3_, 399.777 MHz): δ = 3.68 (*s*, 48H, CH_2_-18-crown-6), 3.58 (*s*, 4H, H_2_O_eq_—Ni), 3.52 (*s*, 4H, H_2_O_eq_—Ni). ^13^C NMR (CDCl_3_, 399.777 MHz): δ = 70.72 (*s*, 18-crown-6). Analysis calculated for C_24_H_64_Cl_2_Ni_1_O_20_: C, 35.93; H, 8.04; N, 0.00. Found: C, 35.97; H, 8.00; N, <0.10.

## 6. Refinement

Crystal data, data collection and structure refinement details are summarized in Table 2 ▸. Hydrogen atoms attached to oxygen were freely refined, and those attached to carbon were refined using a riding model.

## Supplementary Material

Crystal structure: contains datablock(s) I. DOI: 10.1107/S2056989024010041/oi2011sup1.cif

Structure factors: contains datablock(s) I. DOI: 10.1107/S2056989024010041/oi2011Isup2.hkl

CCDC reference: 2391132

Additional supporting information:  crystallographic information; 3D view; checkCIF report

## Figures and Tables

**Table 1:** Hydrogen-bond geometry (Å, °)

*D*—H⋯*A*	*D*—H	H⋯*A*	*D*⋯*A*	*D*—H⋯*A*
O1—H1*C*⋯O4	0.797 (18)	1.973 (18)	2.7679 (12)	174.7 (17)
O1—H1*D*⋯O6	0.780 (18)	1.956 (18)	2.7360 (12)	177.3 (17)
O2—H2*C*⋯O5^i^	0.79 (2)	1.99 (2)	2.7695 (12)	167 (1)
O2—H2*D*⋯Cl1	0.803 (19)	2.335 (19)	3.1258 (9)	168.6 (16)
O3—H3*C*⋯O10	0.82 (2)	1.84 (2)	2.6229 (12)	160 (2)
O3—H3*D*⋯O7	0.780 (18)	2.146 (18)	2.8819 (11)	157.4 (17)
O10—H10*C*⋯Cl1^ii^	0.83 (2)	2.38 (2)	3.2038 (10)	171 (2)
O10—H10*D*⋯Cl1	0.86 (2)	2.30 (2)	3.1559 (10)	173 (2)

Symmetry codes: (i) 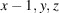; (ii) 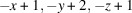.

**Table 2:** Experimental details

Crystal data	
Chemical formula	[Ni(H_2_O)_6_]Cl_2_·2C_12_H_24_O_6_·2H_2_O
*M*_r_	401.18
Crystal system, space group	Triclinic, *P* 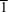
Temperature (K)	105
*a*, *b*, *c* (Å)	7.6472 (2), 10.4180 (3), 12.7214 (3)
α, β, γ (°)	77.288 (1), 77.649 (1), 75.400 (1)
*V* (Å^3^)	943.16 (4)
*Z*	2
Radiation type	Mo *K*α
μ (mm^−1^)	0.73
Crystal size (mm)	0.3 × 0.2 × 0.15

Data collection	
Diffractometer	Bruker D8 Venture Kappa
Absorption correction	Multi-scan (*SADABS*; Krause *et al.*, 2015 ▸)
*T*_min_, *T*_max_	0.708, 0.753
No. of measured, independent and observed [*I* > 2σ(*I*)] reflections	57779, 4152, 4021
*R*_int_	0.030
(sin θ/λ)_max_ (Å^−1^)	0.641

Refinement	
*R*[*F*^2^ > 2σ(*F*^2^)], *wR*(*F*^2^), *S*	0.022, 0.056, 1.05
No. of reflections	4152
No. of parameters	243
H-atom treatment	H atoms treated by a mixture of independent and constrained refinement
Δρ_max_, Δρ_min_ (e Å^−3^)	0.55, −0.27

Computer programs: *APEX4* and *SAINT* (Bruker, 2016 ▸), *SHELXT2014/5* (Sheldrick, 2015*a* ▸), *SHELXL2019/3* (Sheldrick, 2015*b* ▸) and *OLEX2* (Dolomanov *et al.*, 2009 ▸).

## supplementary crystallographic information

### Hexaaquanickel(II) dichloride–1,4,7,10,13,16-hexaoxacyclooctadecane–water (1/2/2) Crystal data

**Table:**

[Ni(H_2_O)_6_]Cl_2_·2C_12_H_24_O_6_·2H_2_O	*Z* = 2
*M**_r_* = 401.18	*F*(000) = 430
Triclinic, *P*1	*D*_x_ = 1.413 Mg m^−^^3^
*a* = 7.6472 (2) Å	Mo *K*α radiation, λ = 0.71073 Å
*b* = 10.4180 (3) Å	Cell parameters from 401129 reflections
*c* = 12.7214 (3) Å	θ = 3.3–61.8°
α = 77.288 (1)°	µ = 0.73 mm^−^^1^
β = 77.649 (1)°	*T* = 105 K
γ = 75.400 (1)°	Prism, blue
*V* = 943.16 (4) Å^3^	0.3 × 0.2 × 0.15 mm

### Hexaaquanickel(II) dichloride–1,4,7,10,13,16-hexaoxacyclooctadecane–water (1/2/2) Data collection

**Table:**

Bruker D8 Ventrue Kappa diffractometer	4021 reflections with *I* > 2σ(*I*)
φ and ω scans	*R*_int_ = 0.030
Absorption correction: multi-scan (SADABS; Krause et al. 2015)	θ_max_ = 27.1°, θ_min_ = 2.8°
*T*_min_ = 0.708, *T*_max_ = 0.753	*h* = −9→9
57779 measured reflections	*k* = −13→13
4152 independent reflections	*l* = −16→16

### Hexaaquanickel(II) dichloride–1,4,7,10,13,16-hexaoxacyclooctadecane–water (1/2/2) Refinement

**Table:**

Refinement on *F*^2^	0 restraints
Least-squares matrix: full	Hydrogen site location: mixed
*R*[*F*^2^ > 2σ(*F*^2^)] = 0.022	H atoms treated by a mixture of independent and constrained refinement
*wR*(*F*^2^) = 0.056	*w* = 1/[σ^2^(*F*_o_^2^) + (0.0229*P*)^2^ + 0.4776*P*] where *P* = (*F*_o_^2^ + 2*F*_c_^2^)/3
*S* = 1.05	(Δ/σ)_max_ = 0.001
4152 reflections	Δρ_max_ = 0.55 e Å^−^^3^
243 parameters	Δρ_min_ = −0.27 e Å^−^^3^

### Hexaaquanickel(II) dichloride–1,4,7,10,13,16-hexaoxacyclooctadecane–water (1/2/2) Special details

**Table:**

Geometry. All esds (except the esd in the dihedral angle between two l.s. planes) are estimated using the full covariance matrix. The cell esds are taken into account individually in the estimation of esds in distances, angles and torsion angles; correlations between esds in cell parameters are only used when they are defined by crystal symmetry. An approximate (isotropic) treatment of cell esds is used for estimating esds involving l.s. planes.

### Hexaaquanickel(II) dichloride–1,4,7,10,13,16-hexaoxacyclooctadecane–water (1/2/2) Fractional atomic coordinates and isotropic or equivalent isotropic displacement parameters (Å^2^)

**Table:**

	*x*	*y*	*z*	*U*_iso_*/*U*_eq_	
Ni1	0.500000	0.500000	0.500000	0.00937 (6)	
O1	0.74883 (11)	0.54896 (9)	0.47148 (7)	0.01664 (16)	
H1C	0.821 (2)	0.5382 (17)	0.4171 (15)	0.030 (4)*	
H1D	0.778 (2)	0.5951 (18)	0.5023 (14)	0.029 (4)*	
O2	0.41319 (11)	0.65045 (8)	0.37574 (6)	0.01457 (15)	
H2C	0.305 (2)	0.6640 (11)	0.3818 (7)	0.022*	
H2D	0.444 (2)	0.7214 (19)	0.3595 (14)	0.033 (4)*	
O3	0.40189 (11)	0.62253 (8)	0.61474 (7)	0.01512 (16)	
H3C	0.408 (3)	0.700 (2)	0.5864 (9)	0.050 (6)*	
H3D	0.405 (2)	0.6015 (17)	0.6772 (15)	0.030 (4)*	
Cl1	0.47188 (4)	0.94579 (3)	0.31034 (2)	0.02239 (7)	
O4	0.98032 (11)	0.51777 (8)	0.27500 (6)	0.01835 (17)	
O5	1.04252 (11)	0.72559 (8)	0.36473 (6)	0.01574 (16)	
O6	0.83961 (11)	0.71618 (8)	0.57969 (7)	0.01876 (17)	
C1	1.04444 (16)	0.38560 (12)	0.24807 (10)	0.0199 (2)	
H1A	1.082780	0.393417	0.167909	0.024*	
H1B	0.942598	0.337326	0.269884	0.024*	
C2	1.08752 (17)	0.61212 (12)	0.21450 (9)	0.0205 (2)	
H2A	1.081962	0.625478	0.135692	0.025*	
H2B	1.217120	0.577741	0.224260	0.025*	
C3	1.01268 (17)	0.74326 (12)	0.25528 (9)	0.0209 (2)	
H3A	1.074762	0.813511	0.208231	0.025*	
H3B	0.879844	0.772696	0.252643	0.025*	
C4	0.95456 (17)	0.84335 (11)	0.41173 (10)	0.0209 (2)	
H4A	0.826966	0.874342	0.397501	0.025*	
H4B	1.020641	0.916732	0.378365	0.025*	
C5	0.95495 (17)	0.80970 (12)	0.53261 (10)	0.0214 (2)	
H5A	1.081248	0.769581	0.547092	0.026*	
H5B	0.908820	0.892442	0.565154	0.026*	
C6	0.79639 (16)	0.69565 (12)	0.69632 (10)	0.0198 (2)	
H6A	0.693210	0.648771	0.720440	0.024*	
H6B	0.755295	0.784474	0.719885	0.024*	
O7	0.36897 (11)	0.62695 (8)	0.84393 (6)	0.01766 (17)	
O8	0.25973 (11)	0.31411 (8)	0.99165 (6)	0.01729 (16)	
O9	−0.03393 (11)	0.18666 (8)	1.05124 (6)	0.01760 (16)	
C7	0.35114 (16)	0.75844 (11)	0.86804 (9)	0.0179 (2)	
H7A	0.447088	0.800913	0.817911	0.021*	
H7B	0.374123	0.748131	0.943456	0.021*	
C8	0.29069 (15)	0.53668 (11)	0.93233 (9)	0.0151 (2)	
H8A	0.155630	0.567191	0.946136	0.018*	
H8B	0.337591	0.534152	0.999674	0.018*	
C9	0.34296 (15)	0.39801 (11)	0.90153 (9)	0.0154 (2)	
H9A	0.296647	0.399490	0.834220	0.018*	
H9B	0.477788	0.365714	0.889178	0.018*	
C10	0.27876 (15)	0.18097 (11)	0.97362 (9)	0.0167 (2)	
H10A	0.406193	0.129700	0.976358	0.020*	
H10B	0.250080	0.183574	0.900792	0.020*	
C11	0.14764 (15)	0.11431 (11)	1.06188 (9)	0.0170 (2)	
H11A	0.162465	0.019455	1.054209	0.020*	
H11B	0.173090	0.115194	1.134794	0.020*	
C12	−0.16698 (16)	0.14810 (11)	1.14179 (9)	0.0174 (2)	
H12A	−0.129468	0.151928	1.210664	0.021*	
H12B	−0.175679	0.054352	1.143799	0.021*	
O10	0.36399 (13)	0.88510 (9)	0.56722 (8)	0.02240 (18)	
H10C	0.419 (3)	0.923 (2)	0.5967 (16)	0.041 (5)*	
H10D	0.398 (3)	0.9071 (19)	0.4982 (17)	0.038 (5)*	

### Hexaaquanickel(II) dichloride–1,4,7,10,13,16-hexaoxacyclooctadecane–water (1/2/2) Atomic displacement parameters (Å^2^)

**Table:**

	*U*^11^	*U*^22^	*U*^33^	*U*^12^	*U*^13^	*U*^23^
Ni1	0.00966 (9)	0.00851 (9)	0.01044 (9)	−0.00317 (6)	−0.00040 (6)	−0.00254 (6)
O1	0.0145 (4)	0.0227 (4)	0.0167 (4)	−0.0104 (3)	0.0035 (3)	−0.0103 (3)
O2	0.0130 (4)	0.0122 (4)	0.0178 (4)	−0.0037 (3)	−0.0030 (3)	0.0002 (3)
O3	0.0218 (4)	0.0117 (4)	0.0116 (4)	−0.0040 (3)	−0.0014 (3)	−0.0025 (3)
Cl1	0.02783 (15)	0.01424 (13)	0.02534 (15)	−0.00744 (11)	−0.00474 (11)	−0.00032 (10)
O4	0.0194 (4)	0.0197 (4)	0.0153 (4)	−0.0040 (3)	0.0016 (3)	−0.0065 (3)
O5	0.0155 (4)	0.0136 (4)	0.0179 (4)	−0.0015 (3)	−0.0043 (3)	−0.0030 (3)
O6	0.0210 (4)	0.0196 (4)	0.0188 (4)	−0.0061 (3)	−0.0045 (3)	−0.0068 (3)
C1	0.0180 (5)	0.0260 (6)	0.0184 (5)	−0.0046 (4)	−0.0010 (4)	−0.0113 (5)
C2	0.0208 (6)	0.0239 (6)	0.0136 (5)	−0.0046 (5)	0.0028 (4)	−0.0021 (4)
C3	0.0258 (6)	0.0182 (5)	0.0151 (5)	−0.0031 (4)	−0.0032 (4)	0.0023 (4)
C4	0.0248 (6)	0.0120 (5)	0.0266 (6)	−0.0040 (4)	−0.0041 (5)	−0.0048 (4)
C5	0.0211 (6)	0.0214 (6)	0.0270 (6)	−0.0080 (5)	−0.0049 (5)	−0.0105 (5)
C6	0.0160 (5)	0.0234 (6)	0.0217 (6)	−0.0035 (4)	−0.0002 (4)	−0.0110 (5)
O7	0.0225 (4)	0.0152 (4)	0.0140 (4)	−0.0077 (3)	0.0035 (3)	−0.0023 (3)
O8	0.0224 (4)	0.0142 (4)	0.0143 (4)	−0.0067 (3)	0.0027 (3)	−0.0032 (3)
O9	0.0151 (4)	0.0186 (4)	0.0157 (4)	−0.0034 (3)	−0.0003 (3)	0.0015 (3)
C7	0.0180 (5)	0.0170 (5)	0.0198 (5)	−0.0081 (4)	0.0007 (4)	−0.0043 (4)
C8	0.0155 (5)	0.0165 (5)	0.0125 (5)	−0.0054 (4)	0.0000 (4)	−0.0006 (4)
C9	0.0153 (5)	0.0174 (5)	0.0126 (5)	−0.0050 (4)	0.0001 (4)	−0.0015 (4)
C10	0.0165 (5)	0.0141 (5)	0.0189 (5)	−0.0023 (4)	−0.0012 (4)	−0.0045 (4)
C11	0.0173 (5)	0.0130 (5)	0.0195 (5)	−0.0017 (4)	−0.0040 (4)	−0.0012 (4)
C12	0.0200 (5)	0.0161 (5)	0.0152 (5)	−0.0072 (4)	0.0005 (4)	−0.0006 (4)
O10	0.0265 (5)	0.0146 (4)	0.0268 (5)	−0.0077 (3)	−0.0014 (4)	−0.0044 (3)

### Hexaaquanickel(II) dichloride–1,4,7,10,13,16-hexaoxacyclooctadecane–water (1/2/2) Geometric parameters (Å, º)

**Table:**

Ni1—O1	2.0310 (8)	C4—C5	1.5007 (17)
Ni1—O1^i^	2.0310 (8)	C5—H5A	0.9900
Ni1—O2	2.0567 (8)	C5—H5B	0.9900
Ni1—O2^i^	2.0567 (8)	C6—H6A	0.9900
Ni1—O3	2.0474 (8)	C6—H6B	0.9900
Ni1—O3^i^	2.0473 (8)	O7—C7	1.4359 (13)
O1—H1C	0.796 (19)	O7—C8	1.4275 (13)
O1—H1D	0.782 (19)	O8—C9	1.4179 (13)
O2—H2C	0.796 (18)	O8—C10	1.4218 (13)
O2—H2D	0.803 (19)	O9—C11	1.4221 (13)
O3—H3C	0.81 (2)	O9—C12	1.4229 (13)
O3—H3D	0.780 (19)	C7—H7A	0.9900
O4—C1	1.4314 (14)	C7—H7B	0.9900
O4—C2	1.4262 (14)	C7—C12^iii^	1.5091 (16)
O5—C3	1.4236 (14)	C8—H8A	0.9900
O5—C4	1.4317 (13)	C8—H8B	0.9900
O6—C5	1.4270 (14)	C8—C9	1.5137 (15)
O6—C6	1.4292 (14)	C9—H9A	0.9900
C1—H1A	0.9900	C9—H9B	0.9900
C1—H1B	0.9900	C10—H10A	0.9900
C1—C6^ii^	1.5113 (17)	C10—H10B	0.9900
C2—H2A	0.9900	C10—C11	1.5082 (15)
C2—H2B	0.9900	C11—H11A	0.9900
C2—C3	1.5013 (17)	C11—H11B	0.9900
C3—H3A	0.9900	C12—H12A	0.9900
C3—H3B	0.9900	C12—H12B	0.9900
C4—H4A	0.9900	O10—H10C	0.83 (2)
C4—H4B	0.9900	O10—H10D	0.86 (2)
			
O1—Ni1—O1^i^	180.0	O6—C5—H5A	110.0
O1—Ni1—O2^i^	88.38 (3)	O6—C5—H5B	110.0
O1^i^—Ni1—O2^i^	91.62 (3)	C4—C5—H5A	110.0
O1—Ni1—O2	91.62 (3)	C4—C5—H5B	110.0
O1^i^—Ni1—O2	88.38 (3)	H5A—C5—H5B	108.4
O1—Ni1—O3^i^	88.95 (3)	O6—C6—C1^ii^	113.46 (9)
O1—Ni1—O3	91.05 (3)	O6—C6—H6A	108.9
O1^i^—Ni1—O3^i^	91.05 (3)	O6—C6—H6B	108.9
O1^i^—Ni1—O3	88.95 (3)	C1^ii^—C6—H6A	108.9
O2—Ni1—O2^i^	180.0	C1^ii^—C6—H6B	108.9
O3—Ni1—O2^i^	87.10 (3)	H6A—C6—H6B	107.7
O3^i^—Ni1—O2	87.10 (3)	C8—O7—C7	113.97 (8)
O3—Ni1—O2	92.90 (3)	C9—O8—C10	113.64 (8)
O3^i^—Ni1—O2^i^	92.90 (3)	C11—O9—C12	113.03 (8)
O3^i^—Ni1—O3	180.0	O7—C7—H7A	108.6
Ni1—O1—H1C	122.3 (12)	O7—C7—H7B	108.6
Ni1—O1—H1D	126.2 (13)	O7—C7—C12^iii^	114.74 (9)
H1C—O1—H1D	109.8 (17)	H7A—C7—H7B	107.6
Ni1—O2—H2C	109.5	C12^iii^—C7—H7A	108.6
Ni1—O2—H2D	123.9 (13)	C12^iii^—C7—H7B	108.6
H2C—O2—H2D	108.9	O7—C8—H8A	110.1
Ni1—O3—H3C	109.5	O7—C8—H8B	110.1
Ni1—O3—H3D	126.3 (13)	O7—C8—C9	108.12 (8)
H3C—O3—H3D	117.7	H8A—C8—H8B	108.4
C2—O4—C1	114.07 (9)	C9—C8—H8A	110.1
C3—O5—C4	111.15 (9)	C9—C8—H8B	110.1
C5—O6—C6	114.59 (9)	O8—C9—C8	105.27 (8)
O4—C1—H1A	109.1	O8—C9—H9A	110.7
O4—C1—H1B	109.1	O8—C9—H9B	110.7
O4—C1—C6^ii^	112.49 (9)	C8—C9—H9A	110.7
H1A—C1—H1B	107.8	C8—C9—H9B	110.7
C6^ii^—C1—H1A	109.1	H9A—C9—H9B	108.8
C6^ii^—C1—H1B	109.1	O8—C10—H10A	110.1
O4—C2—H2A	110.0	O8—C10—H10B	110.1
O4—C2—H2B	110.0	O8—C10—C11	107.97 (9)
O4—C2—C3	108.55 (9)	H10A—C10—H10B	108.4
H2A—C2—H2B	108.4	C11—C10—H10A	110.1
C3—C2—H2A	110.0	C11—C10—H10B	110.1
C3—C2—H2B	110.0	O9—C11—C10	108.25 (9)
O5—C3—C2	109.06 (9)	O9—C11—H11A	110.0
O5—C3—H3A	109.9	O9—C11—H11B	110.0
O5—C3—H3B	109.9	C10—C11—H11A	110.0
C2—C3—H3A	109.9	C10—C11—H11B	110.0
C2—C3—H3B	109.9	H11A—C11—H11B	108.4
H3A—C3—H3B	108.3	O9—C12—C7^iii^	109.84 (9)
O5—C4—H4A	109.9	O9—C12—H12A	109.7
O5—C4—H4B	109.9	O9—C12—H12B	109.7
O5—C4—C5	108.90 (9)	C7^iii^—C12—H12A	109.7
H4A—C4—H4B	108.3	C7^iii^—C12—H12B	109.7
C5—C4—H4A	109.9	H12A—C12—H12B	108.2
C5—C4—H4B	109.9	H10C—O10—H10D	105.9 (18)
O6—C5—C4	108.27 (9)		
			
O4—C2—C3—O5	66.72 (12)	O7—C8—C9—O8	−179.36 (8)
O5—C4—C5—O6	−66.75 (12)	O8—C10—C11—O9	62.92 (11)
C1—O4—C2—C3	−176.61 (9)	C7—O7—C8—C9	−172.00 (9)
C2—O4—C1—C6^ii^	80.37 (12)	C8—O7—C7—C12^iii^	−80.86 (12)
C3—O5—C4—C5	167.84 (9)	C9—O8—C10—C11	−166.27 (9)
C4—O5—C3—C2	−173.18 (9)	C10—O8—C9—C8	174.26 (9)
C5—O6—C6—C1^ii^	−72.73 (12)	C11—O9—C12—C7^iii^	172.32 (9)
C6—O6—C5—C4	−167.84 (9)	C12—O9—C11—C10	−169.24 (9)

Symmetry codes: (i) −*x*+1, −*y*+1, −*z*+1; (ii) −*x*+2, −*y*+1, −*z*+1; (iii) −*x*, −*y*+1, −*z*+2.

### Hexaaquanickel(II) dichloride–1,4,7,10,13,16-hexaoxacyclooctadecane–water (1/2/2) Hydrogen-bond geometry (Å, º)

**Table:**

*D*—H···*A*	*D*—H	H···*A*	*D*···*A*	*D*—H···*A*
O1—H1*C*···O4	0.797 (18)	1.973 (18)	2.7679 (12)	174.7 (17)
O1—H1*D*···O6	0.780 (18)	1.956 (18)	2.7360 (12)	177.3 (17)
O2—H2*C*···O5^iv^	0.79 (2)	1.99 (2)	2.7695 (12)	167 (1)
O2—H2*D*···Cl1	0.803 (19)	2.335 (19)	3.1258 (9)	168.6 (16)
O3—H3*C*···O10	0.82 (2)	1.84 (2)	2.6229 (12)	160 (2)
O3—H3*D*···O7	0.780 (18)	2.146 (18)	2.8819 (11)	157.4 (17)
O10—H10*C*···Cl1^v^	0.83 (2)	2.38 (2)	3.2038 (10)	171 (2)
O10—H10*D*···Cl1	0.86 (2)	2.30 (2)	3.1559 (10)	173 (2)

Symmetry codes: (iv) *x*−1, *y*, *z*; (v) −*x*+1, −*y*+2, −*z*+1.
